# Targeting Inflammation Across the Myocardial Infarction Continuum: Biomarkers, Imaging, and Emerging Therapies

**DOI:** 10.3390/jcm15176615

**Published:** 2026-08-27

**Authors:** Kristi Hoxha, Isabella Maccaferri, Francesco Paparazzo, Alberto Sarti, Giorgio Sciaramenti, Giovanni Camaiti, Pierpaolo Cioci, Cristina Rizza, Renè Tezze, Ludovica Rita Vocale, Elisabetta Tonet, Federico Marchini, Gianluca Campo, Rita Pavasini

**Affiliations:** Cardiology Unit, Azienda Ospedaliero Universitaria di Ferrara, Via Aldo Moro 8, 44124 Ferrara, Italyisabella.maccaferri@edu.unife.it (I.M.); francescopaparazzo1@gmail.com (F.P.); sarticchi@gmail.com (A.S.); camaitigiovanni@gmail.com (G.C.); pierpaolocioci@gmail.com (P.C.); ludovica.vocale@gmail.com (L.R.V.); mrcfrc2@unife.it (F.M.); cmpglc@unife.it (G.C.)

**Keywords:** myocardial infarction, inflammation, residual inflammatory risk, atherosclerosis, biomarkers, cardiac imaging, precision medicine, colchicine, canakinumab, NLRP3 inflammasome

## Abstract

**Background:** Myocardial infarction (MI) remains a leading cause of morbidity and mortality despite major advances in reperfusion and secondary prevention. Inflammation contributes throughout the MI continuum, from atherosclerotic plaque development and destabilization to myocardial injury, adverse ventricular remodeling, and recurrent cardiovascular events. **Objective:** This narrative review summarizes current evidence on inflammation across the MI continuum, focusing on inflammatory biomarkers, cardiovascular imaging, residual inflammatory risk, and emerging anti-inflammatory therapies. **Methods:** We reviewed current evidence on the pathophysiological and clinical relevance of inflammation in MI, with particular emphasis on circulating biomarkers, multimodality imaging, and inflammation-targeted therapeutic strategies. **Results:** High-sensitivity C-reactive protein remains the best-established biomarker of residual inflammatory risk, while interleukin-6, myeloperoxidase, suPAR, and GlycA provide complementary information. Advanced imaging, including coronary computed tomography-derived perivascular fat attenuation index, cardiac magnetic resonance, and positron emission tomography, may further characterize vascular and myocardial inflammation. Clinical trials support inflammation as a potentially modifiable component of cardiovascular risk; however, therapeutic benefit has been inconsistent across inflammatory targets, agents, and clinical settings. Canakinumab and low-dose colchicine have demonstrated cardiovascular benefit in selected secondary-prevention populations, although recent neutral trials highlight heterogeneity across clinical settings. Similarly, IL-6-targeted strategies have yielded mixed results, with the neutral ZEUS trial underscoring that biomarker reduction does not necessarily translate into cardiovascular benefit, while NLRP3-targeted approaches remain investigational. **Conclusions:** Integrating inflammatory biomarkers, multimodality imaging, and targeted therapies may improve risk stratification and support personalized secondary prevention. Further evidence is needed to define optimal patient selection and determine whether biomarker- or imaging-guided anti-inflammatory strategies improve clinical outcomes.

## 1. Introduction

Despite substantial advances in reperfusion strategies and secondary prevention, acute myocardial infarction (AMI) remains a leading cause of cardiovascular morbidity and mortality worldwide [1,2]. Although intensive lipid-lowering therapy and optimal control of traditional cardiovascular risk factors have significantly improved outcomes, many patients continue to experience recurrent ischemic events despite guideline-directed treatment. As increasingly intensive lipid-lowering strategies, including high-intensity statins and PCSK9 inhibition, have substantially reduced LDL-C-related risk, greater attention has focused on inflammation as a potentially modifiable contributor to residual cardiovascular risk. Atherosclerosis is now recognized as a chronic immune–inflammatory disease in which inflammatory pathways contribute to plaque development, destabilization, coronary thrombosis, myocardial injury, and adverse ventricular remodeling. Experimental and clinical evidence has further demonstrated the close interplay between inflammation and thrombosis, providing the biological basis for the concept of residual inflammatory risk, which persists despite optimal lipid lowering and identifies patients at increased risk of recurrent cardiovascular events [3,4,5,6,7]. Increasing recognition of inflammation as a contributor to residual cardiovascular risk has stimulated growing interest in inflammation-targeted strategies after myocardial infarction. Alongside conventional risk factor modification, inflammatory biomarkers, multimodality cardiovascular imaging, and targeted immunomodulatory therapies are emerging as complementary tools for improving risk stratification and personalizing secondary prevention. In this review, we summarize the current understanding of inflammation across the myocardial infarction continuum, from the molecular mechanisms of atherothrombosis to the clinical application of biomarkers, cardiovascular imaging, and anti-inflammatory therapies, highlighting current evidence and future perspectives for precision cardiovascular medicine.

## 2. Materials and Methods

A targeted literature search was conducted in PubMed/MEDLINE to support this narrative review. Articles published in English from database inception to June 15, 2026, were considered, and the search was updated on July 31, 2026, to incorporate newly available evidence. The search strategy included combinations of the following terms: “myocardial infarction”, “acute myocardial infarction”, “inflammation”, “residual inflammatory risk”, “hs-CRP”, “interleukin-6”, “IL-1β”, “NLRP3 inflammasome”, “inflammatory biomarkers”, “atherosclerosis”, “atherothrombosis”, “plaque rupture”, “plaque erosion”, “cardiovascular imaging”, “coronary computed tomography angiography”, “fat attenuation index”, “positron emission tomography”, “cardiac magnetic resonance”, “optical coherence tomography”, “anti-inflammatory therapy”, “colchicine”, “canakinumab”, “tocilizumab”, and “ziltivekimab”. Eligibility was restricted to articles published in English in peer-reviewed journals addressing the mechanisms, assessment, prognostic relevance, imaging, or therapeutic modulation of inflammation across the myocardial infarction continuum. Particular emphasis was placed on landmark randomized clinical trials, observational studies, clinical guidelines, consensus documents, and recent evidence relevant to inflammatory biomarkers, cardiovascular imaging, and inflammation-targeted therapies. Ongoing clinical trials and recently reported trial results were also considered when relevant to emerging therapeutic strategies and unmet clinical needs. Studies were selected based on their clinical relevance, methodological robustness, and contribution to the understanding of inflammation in myocardial infarction. When evidence was heterogeneous or discordant, findings from individual studies were presented according to the specific therapeutic target, patient population, and clinical setting rather than being interpreted as evidence of a uniform treatment effect. Given the narrative nature of the review, no formal systematic review or meta-analysis was performed.

## 3. Pathophysiology of Inflammation in Atherosclerosis and Myocardial Infarction

Inflammation contributes to myocardial infarction through a sequence of interconnected processes that extend from chronic vascular injury to acute coronary thrombosis and post-infarction myocardial repair. In the arterial wall, traditional cardiovascular risk factors promote endothelial dysfunction, lipoprotein retention, innate immune activation, and plaque destabilization, thereby creating the biological substrate for atherothrombosis [8,9,10,11,12,13,14,15,16,17]. After coronary occlusion, the inflammatory response shifts from the vessel wall to the myocardium, where necrotic cardiomyocytes trigger leukocyte recruitment and cytokine release. Although this response is essential for clearance of damaged tissue and initiation of repair, excessive or persistent inflammation may amplify ischemia–reperfusion injury, promote adverse ventricular remodeling, and contribute to heart failure progression [12,17]. Understanding these mechanisms provides the rationale for measuring inflammatory activity through circulating biomarkers and imaging, and for targeting selected inflammatory pathways in secondary prevention.

### 3.1. Inflammation as the Driver of Atherothrombosis

Atherosclerosis is now recognized as a chronic immune–inflammatory disease of the arterial wall rather than a passive process of lipid accumulation. Endothelial dysfunction induced by traditional cardiovascular risk factors—including hypertension, diabetes mellitus, smoking, and hypercholesterolemia—promotes the retention and oxidative modification of low-density lipoproteins (LDLs) within the arterial intima. Oxidized LDL (oxLDL) initiates a local inflammatory response by activating endothelial cells and promoting the recruitment of circulating monocytes, which differentiate into macrophages and progressively accumulate lipids, giving rise to foam cells [8,9,10,11].

A key link between lipid accumulation and inflammation is the activation of the NLRP3 inflammasome. Cholesterol crystals, oxidative stress, and other danger-associated molecular patterns (DAMPs) activate this intracellular signaling complex in macrophages, leading to caspase-1 activation and the maturation of interleukin (IL)-1β. IL-1β subsequently induces IL-6 production, amplifying the inflammatory response and stimulating hepatic synthesis of C-reactive protein (CRP). The NLRP3/IL-1β/IL-6 signaling axis has therefore emerged as a central pathway in atherosclerotic disease and represents the biological rationale for several targeted anti-inflammatory therapies currently under investigation [12,13,14].

**Figure 1:**
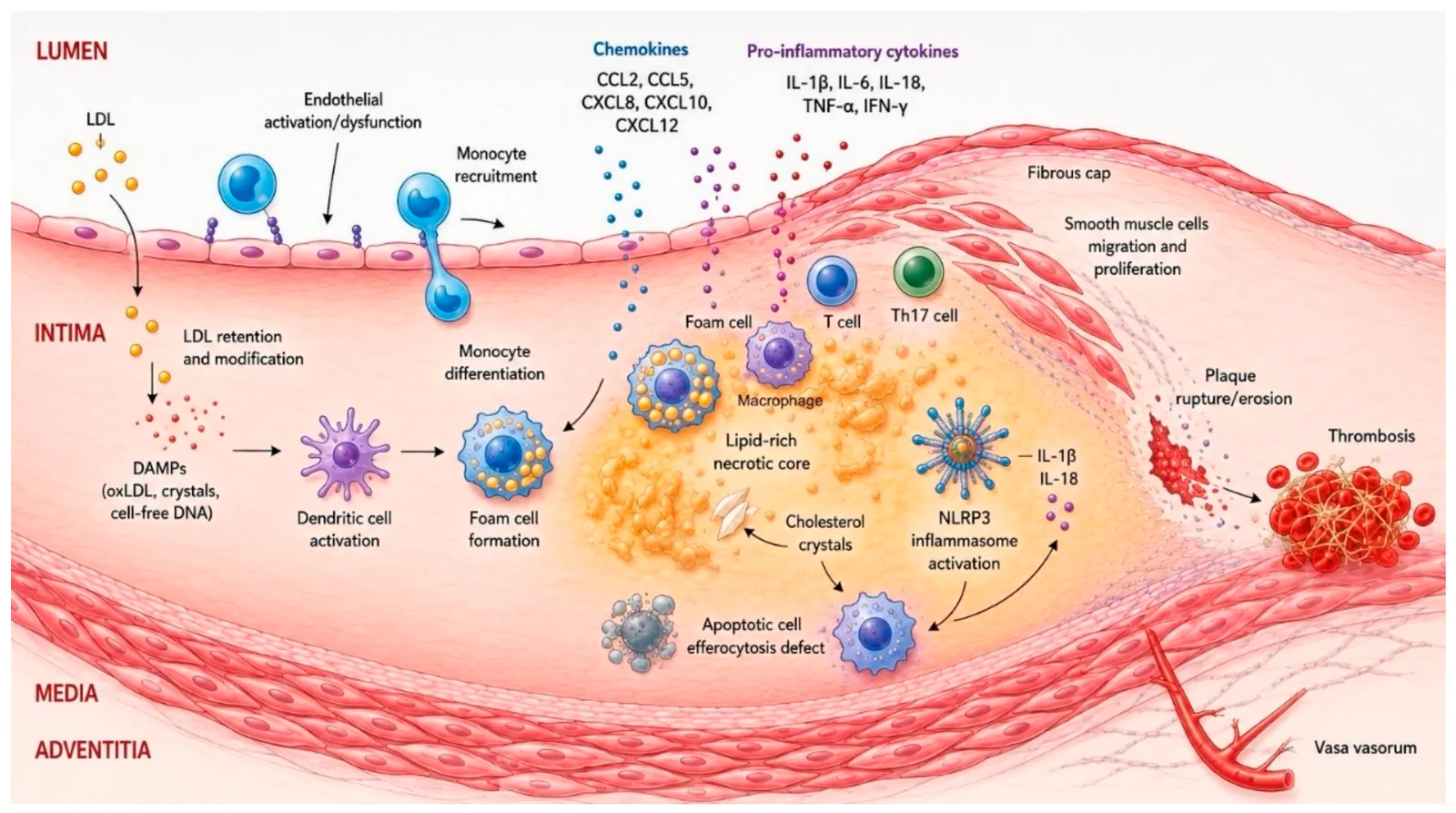
Cellular and molecular mechanisms linking vascular inflammation to plaque vulnerability and coronary thrombosis. Retention and oxidative modification of low-density lipoproteins (LDLs) within the arterial intima generate damage-associated molecular patterns (DAMPs), promoting endothelial activation, leukocyte recruitment, and differentiation of monocytes into macrophages and foam cells. Progressive lipid accumulation, defective efferocytosis, and cholesterol crystal formation activate the NLRP3 inflammasome, leading to the release of pro-inflammatory cytokines, including IL-1β and IL-18, and amplification of local immune responses through chemokines and T-cell activation. Smooth muscle cell migration and fibrous cap remodeling determine plaque stability, whereas persistent inflammation favors fibrous cap thinning, plaque rupture or erosion, and subsequent coronary thrombosis. CCL—C-C motif chemokine ligand; CXCL—C-X-C motif chemokine ligand; DAMPs—damage-associated molecular patterns; IFN-γ—interferon gamma; IL—interleukin; LDL—low-density lipoprotein; NLRP3—nucleotide-binding oligomerization domain-, leucine-rich repeat-, and pyrin domain-containing protein 3; oxLDL—oxidized low-density lipoprotein; Th17—T helper 17; TNF-α—tumor necrosis factor alpha.

Persistent inflammation also determines plaque vulnerability. Activated macrophages promote degradation of the extracellular matrix through matrix metalloproteinases, resulting in thinning of the fibrous cap and expansion of the lipid-rich necrotic core. These features increase the likelihood of plaque rupture, exposing thrombogenic material to the bloodstream and triggering platelet activation and coronary thrombosis [11,15]. Although plaque rupture remains the predominant mechanism underlying acute myocardial infarction, plaque erosion represents an alternative substrate, particularly in younger individuals, women, and smokers, where endothelial injury and neutrophil-mediated inflammation appear to play a more prominent role than extensive lipid accumulation [16] (Figure 1).

### 3.2. Sex-Related Differences in Inflammatory Mechanisms of Myocardial Infarction

Sex-related differences may influence the mechanisms linking inflammation, coronary pathology, and myocardial infarction. Compared with men, women—particularly at younger ages—more frequently present with plaque erosion rather than rupture and have a higher prevalence of non-obstructive and functional coronary mechanisms, including coronary microvascular dysfunction and vasospasm [16,17,18]. Hormonal status may contribute to these differences. Estrogen exerts endothelial-protective and immunomodulatory effects, including enhancement of nitric oxide bioavailability and modulation of vascular inflammatory responses; loss of these protective mechanisms after menopause may promote endothelial and microvascular dysfunction and increase susceptibility to abnormal coronary vasomotion [17]. These sex-related mechanisms may be particularly relevant in myocardial infarction occurring without classic lipid-rich plaque rupture and in type 2 myocardial infarction, in which women are disproportionately represented [19]. Accordingly, sex and hormonal status may contribute to heterogeneity in inflammatory and vascular mechanisms across the myocardial infarction continuum. However, current evidence does not establish a distinct sex-specific inflammatory phenotype or support sex-based selection of anti-inflammatory therapy, highlighting the need for greater representation of women and prespecified sex-specific analyses in future mechanistic and therapeutic studies.

Despite these pathophysiological differences, current evidence does not support different anti-inflammatory treatment strategies according to plaque substrate. Available randomized trials have generally selected patients according to clinical presentation or systemic inflammatory risk rather than plaque morphology. Thus, whether plaque erosion and plaque rupture respond differently to targeted anti-inflammatory therapies remains unknown. Future studies integrating intracoronary imaging with inflammatory biomarkers may clarify whether plaque phenotype can contribute to treatment selection.

### 3.3. From Myocardial Injury to Ventricular Remodeling

Once coronary occlusion occurs, the inflammatory process shifts from the vessel wall to the infarcted myocardium. Following coronary occlusion, myocardial ischemia triggers a rapid and highly coordinated innate immune response. Necrotic cardiomyocytes release DAMPs, including ATP, mitochondrial DNA, and heat-shock proteins, which activate toll-like receptors and inflammasome signaling pathways. This leads to the recruitment of neutrophils and inflammatory monocytes into the infarcted myocardium, where they promote the clearance of necrotic debris and initiate tissue repair [20].

The inflammatory response following myocardial infarction is characterized by a finely regulated temporal sequence. During the early inflammatory phase, neutrophils and pro-inflammatory macrophages dominate the infarct zone, releasing cytokines, proteolytic enzymes, and reactive oxygen species that facilitate debris removal. This phase is subsequently followed by a reparative response, characterized by macrophage polarization toward a pro-resolving phenotype, fibroblast activation, extracellular matrix deposition, and scar formation, all of which are essential for maintaining ventricular integrity.

Failure to appropriately resolve inflammation, however, may shift this physiological response toward maladaptive remodeling. Persistent activation of inflammatory pathways sustains cytokine production, extracellular matrix degradation, and progressive loss of viable myocardium, ultimately promoting left ventricular dilatation, fibrosis, and heart failure. Reperfusion, although indispensable for myocardial salvage, may further amplify these mechanisms through abrupt oxidative stress, endothelial dysfunction, complement activation, and additional NLRP3 inflammasome activation, contributing to microvascular obstruction and ischemia–reperfusion injury [12,20].

Overall, post-infarction inflammation represents a double-edged sword: while an effective inflammatory response is indispensable for tissue repair, excessive or prolonged immune activation contributes to adverse ventricular remodeling and worse clinical outcomes. This delicate balance provides the biological rationale for therapeutic strategies aimed at modulating, rather than completely suppressing, inflammation after myocardial infarction.

### 3.4. Circulating Biomarkers of Inflammatory Risk

Circulating inflammatory biomarkers provide a non-invasive assessment of the biological processes underlying atherosclerotic activity, myocardial injury, and post-infarction remodeling. Although several biomarkers have demonstrated prognostic value, their level of clinical validation differs substantially. High-sensitivity C-reactive protein (hs-CRP) remains the most extensively studied and clinically validated marker of residual inflammatory risk, whereas other biomarkers, including IL-6, myeloperoxidase (MPO), soluble urokinase plasminogen activator receptor (suPAR), and glycoprotein acetylation (GlycA), are emerging as complementary indicators of specific inflammatory pathways [14,21,22,23,24,25,26] (Table 1).

Among these, hs-CRP is currently the most robust biomarker for cardiovascular risk stratification. As the downstream product of IL-6 signaling, hs-CRP reflects systemic inflammatory activity and has been consistently associated with adverse left ventricular remodeling, recurrent ischemic events, and long-term mortality after myocardial infarction. Importantly, clinical trials have shown that selective targeting of specific inflammatory pathways can reduce cardiovascular events in selected populations; however, reductions in inflammatory biomarkers such as hs-CRP have not consistently translated into improved clinical outcomes across therapeutic strategies [14,22,27,28] (Table 1).

Beyond hs-CRP, several biomarkers provide complementary pathophysiological information. IL-6 represents a key upstream mediator of the inflammatory cascade and has emerged as both a prognostic biomarker and a promising therapeutic target. MPO reflects neutrophil activation and oxidative stress and has been associated with plaque instability, microvascular obstruction, and larger infarct size following reperfusion therapy. Similarly, suPAR and GlycA have shown consistent associations with chronic immune activation, endothelial dysfunction, recurrent cardiovascular events, and heart failure, although their routine clinical use remains limited by the lack of standardized cut-off values and prospective validation; for suPAR, the influence of renal function represents an additional consideration when interpreting circulating levels [24,25,26].

Overall, circulating inflammatory biomarkers have expanded the ability to characterize residual inflammatory risk beyond traditional cardiovascular risk factors. However, hs-CRP measurement has not yet been systematically incorporated into routine post-MI care, and its optimal use for guiding therapeutic decisions remains to be established.

## 4. Imaging of Coronary and Myocardial Inflammation

Although circulating biomarkers provide valuable information on systemic inflammatory activity, they cannot localize the site or extent of inflammation. Advanced cardiovascular imaging complements biomarker assessment by enabling non-invasive characterization of vascular and myocardial inflammation throughout the myocardial infarction continuum. Beyond defining coronary anatomy or infarct size, contemporary imaging techniques can identify features of plaque vulnerability, detect inflammatory activity within the vessel wall and the myocardium, and assess the structural consequences of acute myocardial injury. Together, these complementary approaches may improve risk stratification, refine patient selection for anti-inflammatory therapies, and facilitate monitoring of treatment response [20,29].

### 4.1. Positron Emission Tomography: Molecular Imaging of Inflammation

Positron emission tomography (PET) is the reference molecular imaging technique for the assessment of vascular inflammation. The most widely used tracer, the glucose analogue 18F-fluorodeoxyglucose (18F-FDG), accumulates in activated macrophages owing to their increased glycolytic activity, enabling visualization of inflammatory processes within atherosclerotic plaques and infarcted myocardium [30].

However, physiological myocardial glucose uptake limits the evaluation of coronary inflammation with 18F-FDG. This limitation has stimulated the development of more specific tracers targeting inflammatory pathways. Among these, 68Ga-DOTATATE binds somatostatin receptor subtype 2 expressed by activated macrophages, whereas 68Ga-pentixafor targets the CXCR4, a chemokine receptor involved in leukocyte recruitment after MI. These emerging tracers provide greater specificity for imaging inflammatory cell activity [31,32] and may improve patient selection for inflammation-targeted therapies as well as treatment monitoring (Table 2).

### 4.2. Coronary CT Angiography and Perivascular Fat Attenuation Index

Coronary computed tomography angiography (CCTA) has evolved beyond the anatomical assessment of coronary stenosis to provide insights into coronary inflammation through the evaluation of perivascular adipose tissue (PVAT).

Inflammatory signals originating from the vessel wall modify the composition of the surrounding PVAT, by reducing lipid accumulation and increasing aqueous content, resulting in measurable changes in CT attenuation. These changes are quantified by the Perivascular Fat Attenuation Index (FAI), an imaging biomarker that reflects local coronary inflammation [33].

The concept of FAI was first introduced by Antonopoulos et al., who demonstrated that PVAT acts as a dynamic sensor of vascular inflammation and that CT-derived FAI mirrors inflammatory changes within the adjacent artery [33]. Its prognostic value was subsequently confirmed by the landmark CRISP-CT (Cardiovascular RISk Prediction using Computed Tomography) study, where increased FAI independently predicts cardiac mortality beyond traditional cardiovascular risk factors, coronary artery calcium score, stenosis severity, and adverse plaque characteristics [34]. Notably, FAI identifies patients with residual inflammatory risk, even in the absence of high-risk plaque morphology, supporting the concept that vascular inflammation provides prognostic information beyond plaque morphology alone [35].

In addition to inflammation assessment, CCTA enables comprehensive plaque characterization by identifying high-risk morphological features such as positive remodeling, low-attenuation plaque, spotty calcifications, and the napkin-ring sign. The integration of anatomical plaque features with FAI-derived inflammatory information provides a more comprehensive assessment of coronary risk than anatomical imaging alone and may improve identification of patients who could benefit from targeted preventive strategies [35,36] (Table 2).

### 4.3. Cardiac Magnetic Resonance Imaging

Cardiac magnetic resonance (CMR) is the reference imaging modality for myocardial tissue characterization after AMI, providing comprehensive assessment of the structural consequences of ischemic injury and the subsequent inflammatory response. Through multiparametric tissue characterization, CMR enables non-invasive evaluation of myocardial edema, necrosis, fibrosis, and ventricular remodeling, all of which carry important diagnostic and prognostic information [37,38].

T2-weighted imaging and T2 mapping identify myocardial edema, reflecting both the area at risk and the acute inflammatory response following ischemia [37]. Native T1 mapping and extracellular volume (ECV) quantification provide additional information on diffuse myocardial injury and interstitial expansion, whereas late gadolinium enhancement (LGE) remains the gold standard for identifying irreversible necrosis and replacement fibrosis. Beyond tissue characterization, CMR also enables quantification of the myocardial salvage index (MSI), an imaging biomarker that reflects the proportion of myocardium rescued by timely reperfusion. MSI is calculated by relating infarct size, typically assessed by LGE, to the area at risk identified by T2-based edema imaging. A higher MSI has consistently been associated with smaller infarct size, reduced microvascular obstruction, less adverse left ventricular remodeling, and improved long-term prognosis, whereas a lower MSI identifies patients at increased risk of heart failure and recurrent cardiovascular events. As MSI integrates both ischemic injury and the effectiveness of reperfusion therapy, it has emerged as a valuable surrogate endpoint in cardioprotection trials and an important imaging biomarker for evaluating therapeutic strategies aimed at limiting myocardial damage and post-infarction inflammation [37,39,40,41] (Table 2).

By integrating these complementary tissue markers, including edema, infarct size, ECV and myocardial salvage, multiparametric CMR offers a comprehensive evaluation of infarct severity and myocardial repair, improving risk stratification and prediction of adverse ventricular remodeling and long-term clinical outcomes [37,38].

### 4.4. Imaging Plaque Vulnerability and Biology

Contemporary cardiovascular imaging extends beyond the assessment of coronary stenosis to characterize the biological features associated with plaque instability. Rather than focusing solely on the degree of luminal narrowing, current imaging techniques aim to identify plaques with morphological and inflammatory characteristics that predispose to rupture or erosion and, ultimately, to acute coronary events.

CCTA allows non-invasive identification of adverse plaque characteristics. Invasive intracoronary imaging techniques, particularly optical coherence tomography (OCT) and intravascular ultrasound (IVUS) provide high-resolution assessment of plaque microstructure, enabling evaluation of fibrous cap thickness, lipid-rich necrotic core, macrophage infiltration, cholesterol crystals, and plaque erosion [42].

Hybrid PET/CT imaging further integrates anatomical and molecular information by combining plaque morphology with direct assessment of inflammatory activity. This multimodal approach may identify biologically active plaques before structural disruption occurs, offering the potential to improve risk stratification and guide future preventive interventions [29,30] (Table 2).

### 4.5. Future Perspectives

Future developments in cardiovascular imaging are likely to rely on the integration of molecular, anatomical, and functional information to provide a more comprehensive assessment of inflammatory activity across the myocardial infarction continuum. Rather than replacing circulating biomarkers, advanced imaging techniques are expected to complement them by localizing inflammation, characterizing plaque biology, and assessing myocardial injury, thereby enabling a more refined evaluation of residual inflammatory risk [20,34].

Despite these promising advances, several challenges remain. Most inflammation-targeted imaging biomarkers require further technical standardization, validation in prospective studies, and demonstration of incremental clinical value beyond established risk stratification tools. Whether imaging-guided selection of patients for anti-inflammatory therapies improves clinical outcomes also remains to be established.

As evidence continues to evolve, multimodality imaging is expected to play an increasingly important role in precision cardiovascular medicine, supporting individualized risk assessment, therapeutic decision-making, and monitoring of treatment response. No single imaging modality captures the entire inflammatory spectrum. PET offers molecular specificity, CMR characterizes myocardial injury, whereas CCTA identifies coronary inflammation and vulnerable plaque morphology. Their complementary integration with circulating biomarkers may represent the most promising strategy for individualized inflammatory phenotyping.

From a pragmatic and cost-conscious perspective, hs-CRP may represent the most accessible first-line tool for identifying patients with possible residual inflammatory risk. Advanced imaging should not currently be considered a routine second-line test solely on the basis of elevated hs-CRP. Rather, CCTA-derived FAI or PET may be reserved for selected patients in whom further characterization of vascular inflammation could provide additional information beyond conventional clinical assessment, particularly in specialized centers or research settings. Whether such a biomarker-to-imaging step-up strategy improves risk stratification, therapeutic selection, or cost-effectiveness remains to be established in prospective studies.

## 5. Residual Inflammatory Risk and Patient Phenotyping

Despite intensive lipid-lowering therapy and contemporary secondary prevention strategies, a substantial proportion of patients remain at high risk of recurrent cardiovascular events after myocardial infarction. This observation has led to the concept of residual inflammatory risk (RIR), defined as persistent low-grade inflammation despite optimal control of low-density lipoprotein cholesterol (LDL-C), most commonly identified by hs-CRP levels ≥ 2 mg/L [43].

Recent evidence suggests that RIR is highly prevalent in routine clinical practice. A systematic review and meta-analysis of statin-treated patients undergoing contemporary percutaneous coronary intervention (PCI) reported persistent RIR in more than 40% of patients one month after the index event, although this prevalence is likely underestimated because hs-CRP is not routinely assessed in clinical practice [43,44,45]. Importantly, persistent inflammatory activation has emerged as an independent predictor of recurrent major adverse cardiovascular events (MACEs) and all-cause mortality, even among patients achieving guideline-recommended LDL-C targets. As lipid-related risk continues to decline with high-intensity statins and PCSK9 inhibitors, the relative contribution of inflammation to residual cardiovascular risk becomes increasingly evident, highlighting the need for complementary therapeutic strategies [43]. The clinical relevance of RIR extends beyond patients with obstructive coronary artery disease. Elevated inflammatory burden has also been associated with adverse outcomes in myocardial infarction with non-obstructive coronary arteries (MINOCAs), particularly in patients with otherwise well-controlled metabolic risk factors, further supporting inflammation as an independent determinant of prognosis across the spectrum of ischemic heart disease [46].

### 5.1. Residual Cardiovascular Risk: Beyond LDL Cholesterol

Residual cardiovascular risk after MI is multifactorial and reflects the interaction of three closely interconnected components: residual inflammatory, lipid, and thrombotic risk. Although these mechanisms are often considered separately, they represent different aspects of the same pathological process and frequently coexist within the same patient. Residual lipid risk is primarily driven by persistent atherogenic lipoproteins, including triglyceride-rich lipoproteins, remnant cholesterol, and lipoprotein(a). These particles promote lipid retention within the arterial wall, sustaining vascular inflammation and contributing to plaque progression despite optimal LDL-C levels [47]. Residual thrombotic risk reflects persistent platelet activation and enhanced thrombin generation despite antithrombotic therapy. Inflammatory cytokines promote tissue factor expression and amplify coagulation, while activated platelets further enhance leukocyte recruitment and vascular inflammation, establishing a bidirectional relationship between inflammation and thrombosis [48,49]. Ultimately, these interconnected mechanisms favor recurrent plaque disruption and thrombus formation, increasing the risk of recurrent ischemic events [50,51]. Rather than representing competing mechanisms, residual inflammatory, lipid, and thrombotic risk should therefore be viewed as complementary components of residual cardiovascular risk, each potentially requiring a different therapeutic strategy.

### 5.2. Residual Inflammatory and Lipid-Related Risk

Residual cardiovascular risk after myocardial infarction is heterogeneous and may reflect several incompletely controlled biological processes despite guideline-directed secondary prevention. Among these, residual inflammatory risk and residual lipid-related risk represent clinically relevant and potentially complementary components of risk. In addition, plaque-related and thrombotic mechanisms may contribute to recurrent cardiovascular events, underscoring the multifactorial nature of residual risk [52,53].

Residual inflammatory risk is commonly identified by persistently elevated hs-CRP despite adequate control of LDL-C. In contemporary statin-treated populations, elevated hs-CRP has been independently associated with subsequent cardiovascular events and mortality, supporting inflammation as an important contributor to residual risk [14,15]. Conversely, residual lipid-related risk reflects cardiovascular risk associated with persistently elevated atherogenic lipoproteins despite contemporary lipid-lowering therapy. Importantly, inflammatory and lipid-related risk may coexist within the same patient rather than representing mutually exclusive or formally validated phenotypes.

Contemporary secondary-prevention trials have demonstrated reductions in cardiovascular events with intensified lipid-lowering therapy, triglyceride-lowering strategies, and dual-pathway antithrombotic inhibition in appropriately selected populations [54,55,56]. However, these studies did not prospectively assign treatment according to inflammatory or lipid-related risk phenotypes and therefore should not be interpreted as validating a phenotype-guided therapeutic strategy. Similarly, no randomized evidence currently demonstrates that combined lipid-lowering and anti-inflammatory treatment selected according to these residual-risk categories provides greater clinical benefit than either strategy alone. Accordingly, residual inflammatory and lipid-related risk should currently be regarded as complementary dimensions of risk characterization rather than as validated phenotypes for therapeutic selection.

## 6. Therapeutic Targets and Clinical Trials

The recognition of inflammation as an important contributor to residual cardiovascular risk has provided a strong rationale for evaluating inflammation-targeted therapies after myocardial infarction. Selected clinical trials have demonstrated that inhibition of specific inflammatory pathways can reduce recurrent cardiovascular events in defined patient populations, providing proof of concept for this therapeutic approach. However, heterogeneous and neutral findings across different agents and clinical settings indicate that clinical benefit cannot be inferred from inflammatory pathway inhibition or biomarker reduction alone [14,22,27,57,58].

It should also be acknowledged that several established cardiovascular therapies may exert anti-inflammatory effects in addition to their primary mechanisms of action. Statins reduce hs-CRP, as demonstrated in PROVE-IT TIMI 22 and JUPITER, with reductions in inflammatory activity occurring alongside their lipid-lowering effects [45,59]. Bempedoic acid has similarly been associated with reductions in hs-CRP in the CLEAR Outcomes program [60]. In addition, SGLT2 inhibitors and GLP-1 receptor agonists have been associated with modulation of inflammatory biomarkers in randomized trials and meta-analyses [61,62]. These observations suggest that modulation of inflammation may accompany the cardiovascular effects of several established preventive therapies. However, the extent to which these anti-inflammatory effects directly mediate cardiovascular benefit remains uncertain and should be distinguished from therapies specifically designed to target defined inflammatory pathways.

### 6.1. Established Anti-Inflammatory Therapies

Canakinumab was the first therapy to provide clinical proof that selective inhibition of inflammation improves cardiovascular outcomes. In the CANTOS (Canakinumab ANti-inflammatory Thrombosis Outcomes Study) trial, patients with previous myocardial infarction and persistent residual inflammatory risk experienced a significant reduction in recurrent cardiovascular events following IL-1β inhibition, independently of LDL-C reduction [14]. Subsequent studies have provided heterogeneous evidence regarding the cardiovascular efficacy of low-dose colchicine. In COLCOT, initiated within 30 days after myocardial infarction, colchicine significantly reduced the composite risk of cardiovascular death, resuscitated cardiac arrest, myocardial infarction, stroke, or urgent hospitalization for angina leading to coronary revascularization [22]. Similarly, LoDoCo2 demonstrated a significant reduction in cardiovascular events among patients with chronic coronary disease [57]. Conversely, the COPS trial did not demonstrate a significant reduction in its primary cardiovascular endpoint at 12 months in patients with acute coronary syndrome and reported a higher rate of all-cause mortality in the colchicine group [58]. More recently, CLEAR SYNERGY (OASIS 9), the largest randomized evaluation of colchicine in acute myocardial infarction to date, showed no significant reduction in the composite of cardiovascular death, recurrent myocardial infarction, stroke, or ischemia-driven revascularization despite a reduction in C-reactive protein levels [27]. Collectively, these findings suggest that the clinical benefit of colchicine may depend on patient population, clinical setting, and timing of treatment, and do not support its indiscriminate use in the early post-MI setting.

Not all anti-inflammatory therapies have proven effective. Low-dose methotrexate failed to reduce inflammatory biomarkers or cardiovascular events in the CIRT (Cardiovascular Inflammation Reduction Trial), highlighting that suppression of inflammation per se is insufficient and that therapeutic efficacy may depend on targeting biologically relevant inflammatory pathways [63] (Table 3).

Consistent with this heterogeneous evidence, current guideline recommendations remain cautious and differ somewhat between societies. The 2023 AHA/ACC guideline for chronic coronary disease assigns colchicine a Class 2b, Level B-R recommendation, indicating that its addition for secondary prevention may be considered to reduce recurrent atherosclerotic cardiovascular events [64]. In contrast, the 2024 ESC guidelines for chronic coronary syndromes recommend that low-dose colchicine (0.5 mg daily) should be considered in patients with atherosclerotic coronary artery disease to reduce myocardial infarction, stroke, and the need for revascularization (Class IIa, Level A) [65]. Nevertheless, in light of the heterogeneous results across clinical settings, including the neutral findings of COPS and CLEAR SYNERGY in acute coronary syndromes and acute myocardial infarction [27,58], the role of colchicine should be interpreted according to the clinical setting and individual patient characteristics rather than as a universally indicated component of early post-MI therapy.

**Table 3:** Anti-inflammatory therapeutic targets and major clinical trials relevant to myocardial infarction and cardiovascular residual inflammatory risk. ACS: acute coronary syndrome; ASCVD: atherosclerotic cardiovascular disease; ASSAIL-MI: ASSessing the effect of Anti-IL-6 treatment in Myocardial Infarction; CANTOS: Canakinumab Anti-inflammatory Thrombosis Outcomes Study; CIRT: Cardiovascular Inflammation Reduction Trial; CKD: chronic kidney disease; CLEAR SYNERGY: Colchicine and Spironolactone in Patients with Myocardial Infarction; COLCOT: Colchicine Cardiovascular Outcomes Trial; COPS: Colchicine in Patients with Acute Coronary Syndrome; CRP: C-reactive protein; DOBERMANN-T: trial evaluating tocilizumab in patients with acute myocardial infarction at risk of cardiogenic shock; hs-CRP: high-sensitivity C-reactive protein; IL: interleukin; MACE: major adverse cardiovascular events; MI: myocardial infarction; NLRP3: nucleotide-binding oligomerization domain-, leucine-rich repeat-, and pyrin domain-containing protein 3; PCI: percutaneous coronary intervention; STEMI: ST-segment elevation myocardial infarction.

Therapeutic Target	Drug	Key Trial	Study Population	Main Findings	Current Status	References
IL-1β	Canakinumab	CANTOS	Prior MI with persistent inflammatory risk (hs-CRP ≥ 2 mg/L)	Reduced recurrent cardiovascular events and provided proof of concept for selective inflammation targeting	Proof of concept	[14]
Microtubules	Colchicine	COLCOT	Recent MI (within 30 days)	Reduced major adverse cardiovascular events in patients with recent MI	Positive outcome trial; post-MI secondary prevention	[22]
Microtubules	Colchicine	LoDoCo2	Chronic coronary disease	Reduced major adverse cardiovascular events in patients with chronic coronary disease	Guideline-supported in selected patients with chronic coronary disease	[57]
Microtubules	Colchicine	COPS	Acute coronary syndrome	No significant reduction in the primary cardiovascular endpoint at 12 months in patients with ACS; higher all-cause mortality observed	Neutral; safety signal	[58]
Microtubules	Colchicine	CLEAR SYNERGY (OASIS 9)	Acute MI following PCI	No significant reduction in cardiovascular death, recurrent MI, stroke, or ischemia-driven revascularization after acute MI despite CRP reduction	Neutral	[27]
Broad immunosuppression	Methotrexate	CIRT	Prior MI or multivessel coronary disease with diabetes or metabolic syndrome	No reduction in inflammatory biomarkers or cardiovascular events	Not recommended	[63]
IL-6 receptor	Tocilizumab	ASSAIL-MI, DOBERMANN-T	Acute STEMI; Acute MI at risk of cardiogenic shock	Reduced systemic inflammation and markers of myocardial injury; clinical outcome benefit not established	Investigational	[66,67,68]
IL-6 ligand	Ziltivekimab	RESCUE	Chronic kidney disease with elevated hs-CRP; not an acute MI population	Marked reduction in inflammatory biomarkers in patients with CKD and systemic inflammation; not an acute MI trial	Phase 2 completed	[69]
IL-6 ligand	Ziltivekimab	ZEUS	ASCVD and chronic kidney disease with systemic inflammation	Reduced free IL-6 and hs-CRP but did not reduce 3-point MACE in patients with ASCVD, CKD, and systemic inflammation	Phase III completed; neutral	[28]
IL-6 ligand	Ziltivekimab	ARTEMIS	Acute MI	Cardiovascular outcome trial in patients following acute MI; results pending; estimated primary completion 14 December 2026	Phase III ongoing	[70]
IL-6 ligand	Ziltivekimab	HERMES	Heart failure with mildly reduced or preserved ejection fraction and systemic inflammation	Cardiovascular outcome trial in patients with heart failure and systemic inflammation; results pending; estimated primary completion 2 July 2027	Phase III ongoing	[71]
NLRP3 inflammasome	Experimental inhibitors	Early-phase studies	Preclinical and selected early-phase cardiovascular populations	Promising preclinical and early clinical results	Investigational	[72,73]

### 6.2. Emerging Therapeutic Targets

Building on the biological rationale provided by IL-1β inhibition, increasing attention has focused on the IL-6 pathway as a potential therapeutic target. Early clinical studies with the IL-6 receptor antagonist tocilizumab demonstrated reductions in systemic inflammation and markers of myocardial injury following acute myocardial infarction, although evidence of long-term clinical benefit remains limited [66,67,68]. Ziltivekimab, a monoclonal antibody targeting the IL-6 ligand, produced marked reductions in hs-CRP and other inflammatory biomarkers in the phase 2 RESCUE trial, which enrolled patients with moderate-to-severe chronic kidney disease and elevated hs-CRP rather than patients with acute myocardial infarction [69]. More recently, topline results from the phase III ZEUS trial showed that, despite clear biological target engagement with reductions in free IL-6 and hs-CRP, ziltivekimab did not reduce three-point MACE compared with placebo in patients with established atherosclerotic cardiovascular disease, chronic kidney disease, and systemic inflammation (HR 0.99, 95% CI 0.88–1.11) [28]. As these findings are currently available only as topline results, full presentation of the trial data at a scientific meeting later in 2026 is awaited to allow a more comprehensive assessment of the efficacy and safety findings. These findings highlights that reduction in circulating inflammatory biomarkers should not be assumed to translate into improved cardiovascular outcomes. Two additional phase III cardiovascular outcome trials of ziltivekimab remain ongoing: ARTEMIS is evaluating patients following acute myocardial infarction, whereas HERMES is evaluating patients with heart failure with mildly reduced or preserved ejection fraction and systemic inflammation [70,71].

Similarly, direct inhibition of the NLRP3 inflammasome has emerged as an attractive upstream strategy. Experimental studies and early clinical experience suggest that NLRP3 inhibition may attenuate ischemia–reperfusion injury, reduce infarct size, and limit adverse ventricular remodeling, although definitive evidence from large randomized trials is not yet available [72,73]. Beyond cytokine inhibition, novel immunomodulatory approaches aim to promote resolution of inflammation rather than simply suppress immune activation. Strategies targeting regulatory T cells, adaptive immune responses, and cell-based therapies represent promising avenues for restoring immune homeostasis and enhancing myocardial repair after infarction [74]. Future anti-inflammatory strategies are likely to rely on biomarker- and imaging-guided patient selection rather than indiscriminate treatment of all post-MI patients (Table 3).

### 6.3. Safety Considerations of Anti-Inflammatory Therapy

The potential cardiovascular benefits of anti-inflammatory therapies must be balanced against treatment-related adverse effects, particularly the risk of infection. In CANTOS, canakinumab was associated with a higher incidence of fatal infection or sepsis, despite reducing recurrent cardiovascular events [14]. Colchicine has generally shown an acceptable safety profile at low doses in cardiovascular trials, although gastrointestinal intolerance, drug interactions, and its use in patients with severe renal or hepatic impairment require consideration [22,57]. Safety profiles may also differ according to the inflammatory pathway targeted; therefore, findings obtained with one anti-inflammatory agent should not be extrapolated to the entire therapeutic class. Careful patient selection and assessment of comorbidities, concomitant therapies, and individual susceptibility to infection remain essential when considering inflammation-targeted treatment.

## 7. Limits of Current Evidence

Despite growing evidence supporting residual inflammation as an important component of cardiovascular risk after myocardial infarction, several barriers still limit the clinical implementation of inflammation-based risk assessment and targeted therapeutic strategies. First, inflammatory biomarkers remain highly dynamic during the acute and early post-infarction phases. Biomarkers such as hs-CRP and IL-6 are influenced by myocardial necrosis, reperfusion injury, and percutaneous coronary intervention, making the timing of assessment critical. Importantly, although CRP elevation during the acute phase carries prognostic information, it primarily reflects the inflammatory response to the index event and should be distinguished from persistent hs-CRP elevation measured after clinical stabilization, which is used to characterize residual inflammatory risk. Current evidence supports reassessment approximately one month after the index event to better identify persistent rather than transient inflammatory activation [44,75]. In patients with borderline or fluctuating hs-CRP values around the commonly used 2 mg/L threshold, a single measurement should not be considered sufficient to define persistent residual inflammatory risk. Repeated assessment under clinically stable conditions, after excluding transient inflammatory triggers such as infection, may help distinguish persistent from transient inflammatory activation. Importantly, hs-CRP should be interpreted within the overall cardiovascular risk profile rather than as an isolated therapeutic threshold, as no validated algorithm currently supports treatment escalation based solely on serial hs-CRP measurements. Moreover, emerging biomarkers—including MPO and the Systemic Immune-Inflammation Index (SII)—lack standardized cut-off values and prospective validation, limiting their integration into routine clinical practice [76,77].

Importantly, changes in inflammatory biomarkers should not, in isolation, be considered surrogate evidence of clinical benefit. The recent neutral findings of the ZEUS trial, despite substantial suppression of the IL-6 pathway and reduction in hs-CRP, highlight a potential dissociation between biological target engagement and cardiovascular outcomes. Therefore, future studies should prioritize hard clinical endpoints and determine whether biomarker changes can reliably identify treatment response rather than assuming that a reduction in inflammatory markers translates into improved prognosis [28].

Second, advanced imaging techniques capable of directly assessing vascular inflammation, such as PET/CT and CCTA-derived perivascular FAI, remain largely confined to specialized centers because of limited availability, cost, and the need for dedicated expertise [74].

**Figure 2:**
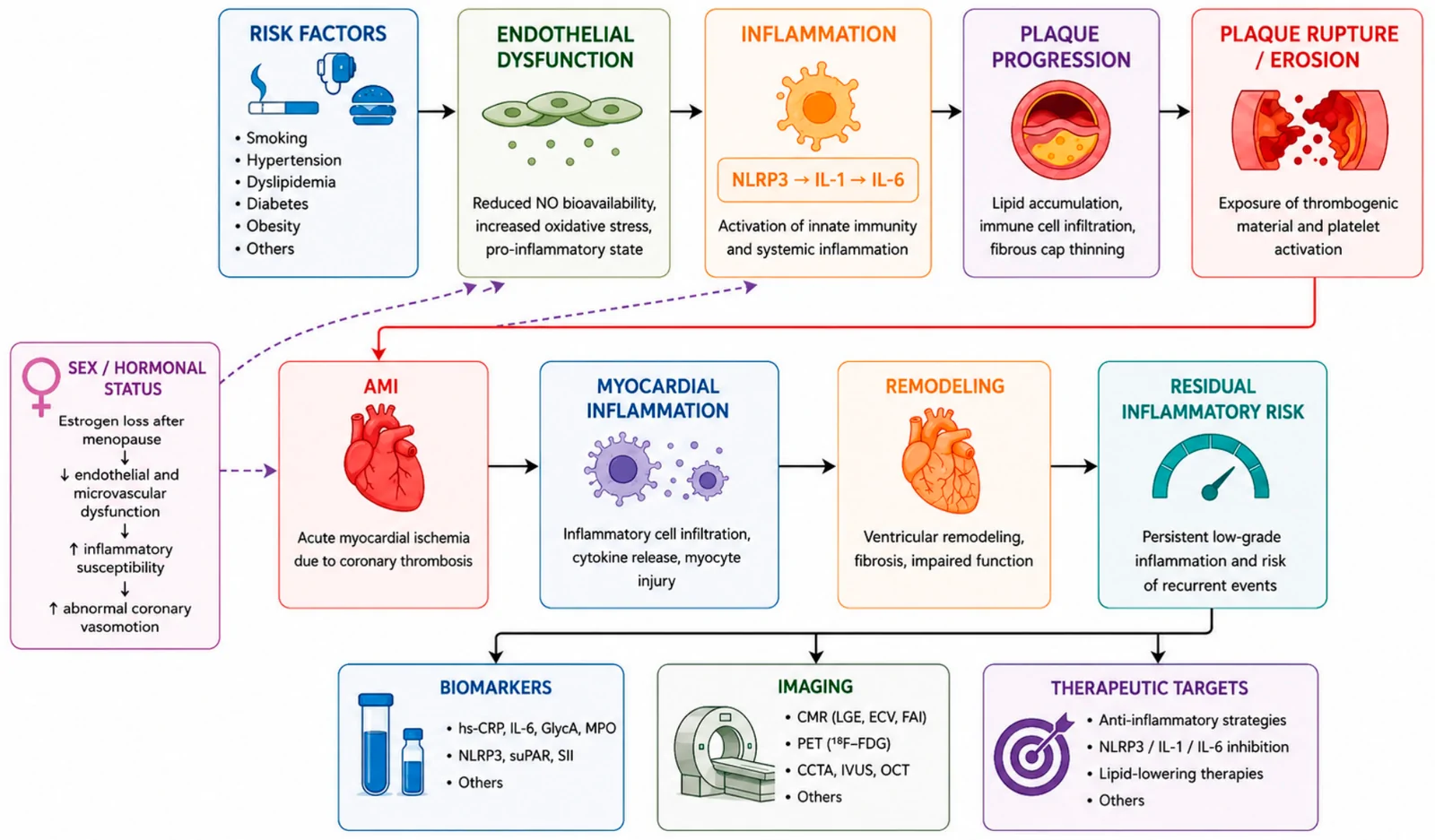
Inflammation across the continuum of atherosclerosis, acute myocardial infarction, and residual inflammatory risk. Traditional cardiovascular risk factors promote endothelial dysfunction, characterized by reduced nitric oxide bioavailability, oxidative stress, and endothelial activation, thereby initiating vascular inflammation. Activation of the NLRP3 inflammasome and the downstream IL-1/IL-6 signaling axis amplifies innate immune responses, driving atherosclerotic plaque progression toward a vulnerable phenotype prone to rupture or erosion. Sex and hormonal status may modulate this continuum through effects on endothelial function, vascular inflammation, microvascular function, and coronary vasomotion, particularly in relation to the loss of estrogen-mediated vascular protection after menopause. Plaque disruption results in acute myocardial infarction (AMI), followed by myocardial inflammation, ventricular remodeling, and persistent residual inflammatory risk, which contributes to recurrent cardiovascular events. Biomarkers of systemic inflammation, multimodality cardiovascular imaging, and emerging anti-inflammatory therapies provide complementary tools for risk stratification and targeted management across the disease spectrum. 18F-FDG—fluorine-18 fluorodeoxyglucose; AMI—acute myocardial infarction; CCTA—coronary computed tomography angiography; CMR—cardiac magnetic resonance; ECV—extracellular volume; FAI—fat attenuation index; GlycA—glycoprotein acetylation; hs-CRP—high-sensitivity C-reactive protein; IL—interleukin; IVUS—intravascular ultrasound; LGE—late gadolinium enhancement; MPO—myeloperoxidase; NLRP3—nucleotide-binding oligomerization domain-, leucine-rich repeat-, and pyrin domain-containing protein 3; NO—nitric oxide; OCT—optical coherence tomography; PET—positron emission tomography; SII—systemic immune-inflammation index; suPAR—soluble urokinase plasminogen activator receptor.

Third, although residual inflammatory risk is increasingly recognized as an important component of secondary prevention, its clinical implementation remains insufficiently standardized. Current guidelines do not provide clear recommendations regarding the optimal biomarker to measure, the most appropriate timing for assessment, or the thresholds required to identify patients most likely to benefit from targeted anti-inflammatory therapies [6,78]. Future studies should therefore focus not only on developing new therapeutic strategies but also on defining practical algorithms integrating biomarkers, imaging, and clinical characteristics to support personalized treatment decisions (Figure 2).

Finally, several limitations of the present review should be acknowledged. Its narrative design and targeted, rather than systematic, literature search may have introduced selection bias and do not ensure exhaustive identification of all relevant studies. In addition, no formal risk-of-bias or quality assessment of the included studies was performed. The evidence discussed was selected primarily on the basis of clinical relevance and contribution to the scope of the review, with particular emphasis on landmark trials, contemporary studies, guidelines, and emerging evidence. Accordingly, the present review should be interpreted as a critical narrative synthesis of the field rather than as a systematic or quantitatively comprehensive assessment of the available evidence.

## 8. Conclusions

Inflammation is now recognized as a central determinant of the entire myocardial infarction continuum, contributing not only to atherosclerotic plaque development and destabilization but also to myocardial injury, ventricular remodeling, and recurrent cardiovascular events. Experimental and clinical evidence supports residual inflammatory risk as an important component of residual cardiovascular risk and provides a rationale for investigating inflammation-targeted therapeutic strategies.

Clinical trials of selective anti-inflammatory therapies have provided proof of concept that modulation of specific inflammatory pathways can reduce cardiovascular events in selected populations beyond conventional lipid lowering. However, this benefit has not been consistently reproduced across inflammatory targets, agents, or clinical settings. In particular, the neutral findings of recent trials of colchicine in acute myocardial infarction and IL-6 ligand inhibition despite reductions in inflammatory biomarkers underscore that biological target engagement does not necessarily translate into improved cardiovascular outcomes [14,22,27,28,57,58]. These findings highlight a central unresolved question: why does modulation of inflammation result in clinical benefit in some therapeutic and patient settings but not in others?

Future research should therefore focus on identifying the inflammatory pathways, patient phenotypes, and timing of intervention most likely to yield clinical benefit, while establishing whether biomarkers or imaging can reliably guide therapeutic selection. Integrating biomarkers, multimodality imaging, and clinical risk profiling may support more refined inflammatory phenotyping; however, biomarker- or imaging-guided treatment algorithms require prospective validation before routine implementation. Thus, inflammation remains a promising but incompletely defined therapeutic domain in secondary prevention after myocardial infarction.

## Figures and Tables

**Table 1:** Emerging inflammatory biomarkers in acute myocardial infarction. GlycA: glycoprotein acetylation; hs-CRP: high-sensitivity C-reactive protein; IL-6: interleukin-6; MPO: myeloperoxidase; suPAR: soluble urokinase plasminogen activator receptor.

Biomarker	Main Pathway	Clinical Significance	Current Status	References
hs-CRP	Systemic inflammation (IL-6–driven acute-phase response)	Residual inflammatory risk assessment; prognosis; patient selection for anti-inflammatory therapies	Clinically validated	[14,21,22]
IL-6	Central inflammatory cytokine	Residual inflammatory risk; prognosis; potential therapeutic target	Phase III outcome trials completed and ongoing	[21,22]
MPO	Neutrophil activation and oxidative stress	Plaque instability, endothelial dysfunction, reperfusion injury	Investigational	[24]
suPAR	Chronic immune activation	Risk stratification for recurrent cardiovascular events and heart failure	Investigational	[26]
GlycA	Chronic systemic inflammation (acute-phase glycoproteins)	Assessment of long-term inflammatory burden and cardiovascular risk	Investigational	[25]

**Table 2:** Imaging modalities for assessment of inflammation after acute myocardial infarction. CCTA: coronary computed tomography angiography; CMR: cardiac magnetic resonance; FAI: fat attenuation index; IVUS: intravascular ultrasound; OCT: optical coherence tomography; PET: positron emission tomography.

Modality	Main Target	Clinical Information	Strength	Limitation	References
PET	Vascular inflammatory activity	Quantification of arterial inflammation and residual inflammatory burden	Molecular imaging; quantitative assessment	Limited availability; high cost	[29,30,31,32]
CCTA/FAI	Coronary perivascular inflammation	Assessment of coronary inflammation and residual inflammatory risk	Non-invasive assessment of coronary anatomy and perivascular inflammation; applicable to routinely acquired CCTA datasets	Radiation exposure; iodinated contrast administration; FAI analysis requires dedicated post-processing software and expertise and is not yet widely available	[33,34,35,36]
CMR	Myocardial tissue characterization	Myocardial edema, fibrosis, infarct size, and ventricular remodeling	Excellent tissue characterization	Limited availability; contraindications in selected patients	[37,38,39,40,41]
OCT/IVUS	Coronary plaque microstructure	Plaque phenotype, vulnerability, and healing	High spatial resolution	Invasive procedure	[42]

## Data Availability

No new data were created or analyzed in this study. Data sharing is not applicable to this article.

## Abbreviations

The following abbreviations are used in this manuscript:

18F-FDG	Fluorine-18 Fluorodeoxyglucose
AMI	Acute Myocardial Infarction
ATP	Adenosine Triphosphate
CCL	C-C Motif Chemokine Ligand
CCTA	Coronary Computed Tomography Angiography
CMR	Cardiac Magnetic Resonance
CRP	C-Reactive Protein
CT	Computed Tomography
CXCL	C-X-C Motif Chemokine Ligand
CXCR4	C-X-C Chemokine Receptor Type 4
DAMPs	Damage-Associated Molecular Patterns
ECV	Extracellular Volume
FAI	Fat Attenuation Index
GlycA	Glycoprotein Acetylation
hs-CRP	High-Sensitivity C-Reactive Protein
IFN-γ	Interferon Gamma
IL	Interleukin
IVUS	Intravascular Ultrasound
LDL	Low-Density Lipoprotein
LDL-C	Low-Density Lipoprotein Cholesterol
LGE	Late Gadolinium Enhancement
MACE	Major Adverse Cardiovascular Events
MINOCA	Myocardial Infarction with Non-Obstructive Coronary Arteries
MPO	Myeloperoxidase
MSI	Myocardial Salvage Index
NLRP3	NOD-, LRR- and Pyrin Domain-Containing Protein 3
NSTEMI	Non-ST-Segment Elevation Myocardial Infarction
OCT	Optical Coherence Tomography
PCI	Percutaneous Coronary Intervention
PCSK9	Proprotein Convertase Subtilisin/Kexin Type 9
PET	Positron Emission Tomography
PVAT	Perivascular Adipose Tissue
RIR	Residual Inflammatory Risk
SII	Systemic Immune-Inflammation Index
STEMI	ST-Segment Elevation Myocardial Infarction
suPAR	Soluble Urokinase Plasminogen Activator Receptor
Th17	T Helper 17
TNF-α	Tumor Necrosis Factor Alpha
